# Functional Outcomes of Hip Fracture Surgery in Elderly Patients at a Tertiary Care Hospital in a Rural Area of North India

**DOI:** 10.7759/cureus.106889

**Published:** 2026-04-12

**Authors:** Ajay Pal Singh Sehrawat, Rajiv Kapila, Kautilya Kautilya, Akhil Sathyan, Vipin Sharma, Vijay Pal Singh, Seema Gupta

**Affiliations:** 1 Department of Orthopaedics, Dr. Rajendra Prasad Government Medical College, Tanda, IND; 2 Department of Anaesthesiology, Dr. Rajendra Prasad Government Medical College, Tanda, IND; 3 Department of Orthodontics, Kothiwal Dental College and Research Centre, Moradabad, IND

**Keywords:** adult, arthroplasty, fracture fixation, hip fracture, internal outcome, replacement

## Abstract

Introduction: Hip fractures in the elderly are associated with significant morbidity, reduced functional independence, and an increased healthcare burden. Functional recovery following surgical management remains a key determinant of patient outcomes, particularly in populations in which daily activities require a higher degree of mobility. This study aimed to evaluate functional outcomes following hip fracture surgery in elderly patients and to compare recovery across fracture types and surgical interventions.

Materials and methods: This prospective observational study included 161 patients aged ≥60 years with proximal femoral fractures that were managed surgically at Dr. Rajendra Prasad Government Medical College (Dr. RPGMC), Kangra, in Tanda, Himachal Pradesh, India. Baseline demographic and clinical data were recorded. Functional outcomes were assessed using the Functional Ambulation Category and Modified Harris Hip Score postoperatively on day one, fourteen days, six weeks, three months, six months, nine months, and one year later. Culturally relevant functional abilities such as squatting and sitting cross-legged were also evaluated. Statistical analysis was performed using repeated measures analysis of variance and chi-square test, with p<0.05 considered significant.

Results: Intertrochanteric fractures were the most common type, followed by femoral neck fractures and subtrochanteric fractures. Closed reduction and internal fixation with proximal femoral nailing was the predominant intervention. Surgery was performed after six days in 113 patients (70.2%). Postoperative complications were observed in three patients (1.9%), and 143 patients (88.8%) survived. Functional Ambulation Category scores improved significantly from the immediate postoperative period to one year (p<0.001), with no significant differences between fracture types or surgical interventions (p>0.05). Similarly, the Modified Harris Hip Score showed significant improvement over time (p<0.001) without significant intergroup differences (p>0.05). However, better recovery of squatting and cross-legged sitting was observed in patients treated with arthroplasty at nine months and one year (p<0.05).

Conclusion: Hip fracture surgery in elderly patients resulted in significant functional improvement over time. While the overall outcomes are comparable across surgical modalities, arthroplasty offers advantages in restoring culturally relevant functional activities.

## Introduction

Hip fractures are among the most serious injuries affecting the elderly population and are associated with significant morbidity, mortality, and loss of independence. With the global rise in life expectancy, the incidence of hip fractures is increasing, particularly in developing countries, such as India [1,2]. These fractures commonly occur due to low-energy trauma to osteoporotic bones and frequently affect individuals above 60 years of age. The burden of hip fractures extends beyond physical disability, contributing to prolonged hospitalization, increased healthcare costs, and a substantial decline in the quality of life [3].

Management of hip fractures primarily involves surgical intervention aimed at early mobilization, pain relief, and restoration of the pre-fracture functional status. Various surgical options, such as internal fixation (such as proximal femoral nailing) and arthroplasty procedures (hemiarthroplasty or total hip replacement), are employed depending on the fracture type and patient characteristics [4,5]. However, functional recovery following surgery is influenced by multiple factors, including age, comorbidities, timing of surgery, rehabilitation, and socio-environmental conditions. In rural and hilly regions, delayed access to healthcare services and limited rehabilitation facilities further complicate recovery outcomes.

Functional outcomes are commonly assessed using validated tools, such as the Functional Ambulation Category (FAC) and Modified Harris Hip Score, which evaluate mobility, pain, and ability to perform daily activities [6,7]. Despite advancements in surgical techniques, many elderly patients fail to regain their pre-injury functional status, highlighting the need for region-specific outcome evaluations. The present study aimed to evaluate functional outcomes following hip fracture surgery in elderly patients. The primary objective of this study was to evaluate functional outcomes following hip fracture surgery in elderly patients. The secondary objectives were to compare functional recovery across different fracture types and surgical interventions and to assess the restoration of culturally relevant functional activities, including squatting and sitting cross-legged, over defined follow-up intervals.

## Materials and methods

This prospective observational cohort study was conducted at Dr. Rajendra Prasad Government Medical College (Dr. RPGMC), Kangra in Tanda, Himachal Pradesh, India, a tertiary care teaching hospital catering to a predominantly rural, hilly population. The study was conducted over a period of one year, from May 2024 to May 2025. Ethical approval was obtained from the Institutional Ethics Committee of Dr. RPGMC, Kangra at Tanda, Himachal Pradesh, prior to commencement of the study (Approval No.: HFW-H DRPGMC/Ethics/2024/57; dated April 27, 2024). The study protocol was in accordance with the ethical principles of the Declaration of Helsinki. Written informed consent was obtained from all participants prior to inclusion in the study.

Sample size estimation was performed using the G Power software (version 3.1; Heinrich Heine University Düsseldorf, Düsseldorf, Germany). Based on repeated-measures analysis of variance for functional recovery outcomes, assuming a moderate effect size (Cohen’s f = 0.25) referencing a previous study [8], significance level of 0.05, and statistical power of 80%, the minimum required sample size was calculated to be 128. Considering the possible attrition rate of 20%, the target sample size was set to 154. Ultimately, 161 patients were included in this study.

Patients were recruited consecutively based on the predefined eligibility criteria. Inclusion criteria comprised patients aged 60 years and above who presented with proximal femoral fractures, including femoral neck, intertrochanteric, and subtrochanteric fractures, and who underwent surgical management. Patients with pathological fractures or polytrauma or those unwilling to provide consent were excluded from the study. Patients were recruited consecutively to minimize selection bias. Eligibility was assessed at the time of admission by the treating orthopedic team based on predefined criteria.

Baseline data, including demographic characteristics, occupation, comorbidities, fracture type, and type of surgical intervention, were recorded. The surgical procedures included closed reduction and internal fixation with proximal femoral nailing, open reduction and internal fixation with proximal femoral nailing, bipolar hemiarthroplasty, and total hip replacement. Perioperative variables, such as time from admission to surgery, duration of hospital stay, postoperative complications, and survival status, were documented. All clinical and functional data were recorded using a standardized data collection pro forma by trained investigators. Follow-up assessments were conducted at predefined intervals either during hospital visits or scheduled outpatient follow-ups to ensure consistency in data recording.

Functional outcomes were assessed using two validated instruments: the Functional Ambulation Category (FAC) and the Modified Harris Hip Score. The Functional Ambulation Category is a six-point ordinal scale ranging from 0 to 5, which is used to assess walking ability based on the level of physical support required [6]. A score of 0 indicates non-functional ambulation, whereas a score of 5 indicates independent ambulation on all surfaces without assistance. 

The Modified Harris Hip Score is a patient-reported outcome measure derived from the original Harris Hip Score that focuses on pain and functional ability [7]. It consists of one domain assessing pain (maximum 44 points) and seven items assessing function (maximum 47 points), including gait parameters such as limp, support, and distance walked, as well as functional activities such as stair climbing, sitting, and footwear use. The total raw score was scaled to a maximum of 100, with higher scores indicating better hip function. Functional assessments were carried out at predefined follow-up intervals on postoperative day one, day fourteen, six weeks, three months, six months, nine months, and one year. In addition, culturally relevant functional abilities such as squatting and sitting cross-legged were evaluated at each follow-up visit. All functional assessments were performed by the same clinical team to reduce inter-observer variability. Standardized instructions were provided to patients during each assessment to maintain uniformity.

Statistical analyses were performed using the IBM Corp. Released 2020. IBM SPSS Statistics for Windows, Version 26. Armonk, NY: IBM Corp. Descriptive statistics are expressed as frequencies and percentages for categorical variables and means with standard deviations for continuous variables. Group comparisons were performed using the Chi-squared test or Fisher’s exact test, as appropriate. Longitudinal changes in functional outcomes were analyzed using mixed-model repeated-measures analysis of variance, with time as a within-subject factor, and fracture type or surgical intervention as between-subject factors. Statistical significance was set at p < 0.05. The cervicotrochanteric fracture group was excluded from inferential statistical analysis due to an insufficient sample size, ensuring the validity and stability of the statistical model. Missing data, if any, were handled using complete-case analysis. Data normality was assessed and confirmed prior to applying parametric tests. All statistical tests were two-tailed.

## Results

Out of 161 patients, 55 (34.2%) patients were aged 60-70 years and 52 (32.3%) were 71-80 years, with a predominance of females. Most participants were homemakers, followed by farmers. Although a substantial proportion had no comorbidities, multiple comorbid conditions were present in nearly one-fourth of the study population. Intertrochanteric fractures were the most frequent fracture type, and closed reduction and internal fixation with proximal femoral nailing were the most commonly performed surgical procedures. A considerable delay in surgical intervention was observed, with the majority undergoing surgery after six days of admission. More than half of the patients had a hospital stay > 10 days. Postoperative complications were rare, and the overall survival rate remained high (Table 1).

**Table 1:** Descriptive analysis of characteristics of study population. Values are expressed as numbers (percentages).

Characteristic	Category	N (%)
Age Group (Years)	60–70	55 (34.2)
	71–80	52 (32.3)
	81–90	37 (23.0)
	>90	17 (10.6)
Sex	Male	52 (32.3)
	Female	109 (67.7)
Occupation	Homemaker / Housemaker	110 (67.7)
	Farmer	47 (29.2)
	Other (Barber, Painter, Retired, Teacher)	5 (3.1)
Medical History (Comorbidities)	No Comorbidities	54 (33.5)
	Hypertension (HTN) Only	29 (18.0)
	HTN and Type 2 Diabetes Mellitus (T2DM)	10 (6.2)
	T2DM Only	5 (3.1)
	Chronic Obstructive Pulmonary Disease (COPD)	3 (1.9)
	Cardiac ailments (CAD, cardiomyopathy)	4 (1.9)
	Hypothyroidism	5 (1.9)
	Rheumatoid Arthritis (RA)	2 (1.2)
	Chronic Kidney Disease (CKD)	3 (1.2)
	Other (Cancer, Psychiatric, etc.)	10 (6.2)
	Multiple Comorbidities (HTN, T2DM, Cardiac ailments, etc.)	40 (24.8)
Fracture Type	Femoral Neck Fracture	32 (19.9)
	Intertrochanteric Fracture	119 (73.9)
	Subtrochanteric Fracture	9 (5.6)
	Cervicotrochanteric Fracture	1 (0.6)
Surgical Intervention	CRIF with PFN	121 (5.2)
	ORIF with PFN	4 (1.9)
	Bipolar Hemiarthroplasty	30 (18.6)
	Total Hip Replacement (THR)	6 (3.7)
Time from Admission to Surgery (Days)	≤2 days	10 (6.2)
	3–5 days	38 (23.6)
	6–10 days	62 (38.5)
	>10 days	51 (31.7)
Hospital Stay Duration (Days)	≤7 days	31 (19.3)
	8–10 days	41 (25.5)
	11–14 days	38 (23.6)
	15–20 days	44 (27.3)
	>20 days	7 (4.3)
Postoperative Complications	None (Nil)	158 (98.1)
	Present	3 (1.2)
Survival Status	Survived	143 (88.8)
	Deceased	18 (11.1)

A comparison of postoperative outcomes across fracture types (Table 2) revealed no significant differences. Time to surgery, duration of hospital stay, complication rates, and survival outcomes were comparable among femoral neck, intertrochanteric, and subtrochanteric fractures, indicating that the fracture type did not significantly influence immediate clinical outcomes.

**Table 2:** Surgical and clinical outcomes by fracture type. Values are expressed as numbers (percentages); the chi-square test was used for comparison between groups. A p-value <0.05 was considered statistically significant.

Outcome	Category	Femoral neck (n=32)	Intertrochanteric (n=119)	Subtrochanteric (n=9)	Chi stats	p-value
Time from admission to surgery (Days)	≤2 days	3 (9.4%)	6 (5.0%)	1 (11.1%)	1.468	0.962
	3–5 days	8 (25.0%)	28 (23.3%)	2 (22.2%)		
	6–10 days	11 (34.4%)	48 (40.0%)	3 (33.3%)		
	>10 days	10 (31.3%)	38 (31.7%)	3 (33.3%)		
duration of hospital stay (Days)	≤7 days	6 (18.8%)	23 (20.0%)	1 (11.1%)	2.239	0.973
	8–10 days	8 (25.0%)	30 (25.0%)	3 (33.3%)		
	11–14 days	8 (25.0%)	28 (23.3%)	2 (22.2%)		
	15–20 days	8 (25.0%)	34 (28.3%)	2 (22.2%)		
	>20 days	2 (6.3%)	4 (3.3%)	1 (11.1%)		
Postoperative complications	Absent	31 (96.9%)	118 (99.2%)	9 (100.0%)	1.187	0.552
	Present	1 (3.1%)	1 (0.8%)	0 (0.0%)		
Survival status	Survived	28 (87.5%)	106 (89.2%)	8 (88.9%)	0.063	0.969
	Deceased	4 (12.5%)	13 (10.8%)	1 (11.1%)		

Similarly, analysis based on surgical intervention (Table 3) demonstrated no statistically significant differences in postoperative parameters across the closed reduction and internal fixation with proximal femoral nailing, open reduction and internal fixation with proximal femoral nailing, bipolar hemiarthroplasty, and total hip replacement groups.

**Table 3:** Surgical and clinical outcomes by surgical intervention. Values are expressed as numbers (percentages); the chi-square test was used for comparison between groups. A p-value <0.05 was considered statistically significant.

Outcome	Category	Closed reduction and internal fixation with proximal femoral nailing (n=121)	Open reduction and internal fixation with proximal femoral nailing (n=4)	Bipolar Hemiarthroplasty (n=30)	Total hip replacement (n=6)	Chi stats	p-value
Time from admission to surgery (Days)	≤2 days	6 (5.0%)	1 (25.0%)	2 (6.7%)	1 (16.7%)	4.722	0.858
	3–5 days	28 (23.1%)	1 (25.0%)	7 (23.3%)	2 (33.3%)		
	6–10 days	48 (39.7%)	1 (25.0%)	11 (36.7%)	2 (33.3%)		
	>10 days	39 (32.2%)	1 (25.0%)	10 (33.3%)	1 (16.7%)		
Hospital Stay duration (Days)	≤7 days	24 (19.8%)	0 (0.0%)	6 (20.0%)	1 (16.7%)	5.422	0.942
	8–10 days	31 (25.6%)	1 (25.0%)	7 (23.3%)	2 (33.3%)		
	11–14 days	28 (23.1%)	1 (25.0%)	8 (26.7%)	1 (16.7%)		
	15–20 days	34 (28.1%)	2 (50.0%)	7 (23.3%)	1 (16.7%)		
	>20 days	4 (3.3%)	0 (0.0%)	2 (6.7%)	1 (16.7%)		
Postoperative complications	Absent	120 (99.2%)	4 (100.0%)	29 (96.3%)	6 (100.0%)	1.366	0.714
	Present	1 (0.8%)	0 (0.0%)	1 (3.3%)	0 (0.0%)		
Survival status	Survived	109 (90.1%)	3 (75.0%)	26 (86.7%)	5 (83.3%)	1.28	0.733
	Deceased	12 (9.9%)	1 (25.0%)	4 (13.3%)	1 (16.7%)		

**Figure 1:**
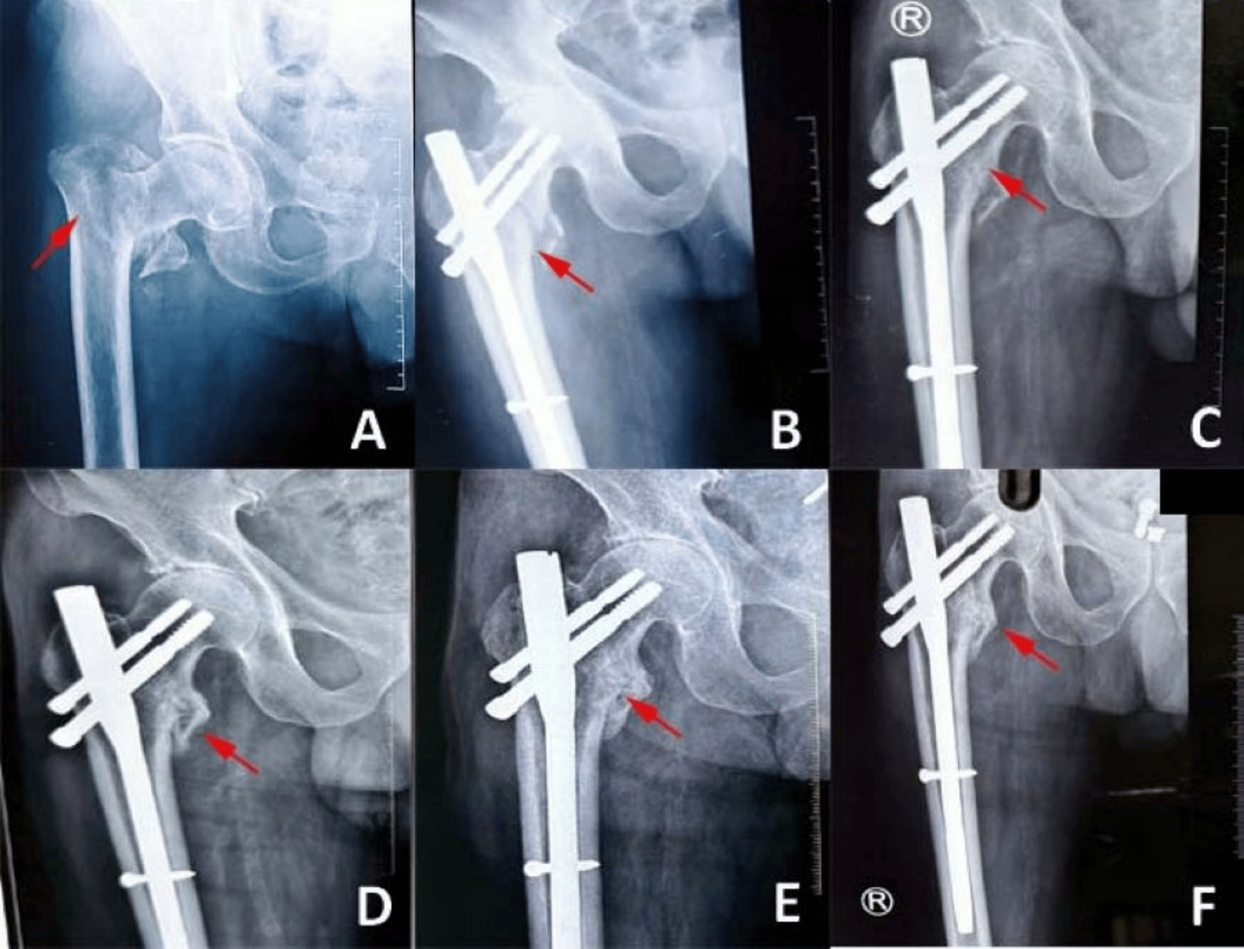
Serial radiographs demonstrating postoperative implant position and fracture healing: (A) Preoperative radiograph showing intertrochanteric fracture; (B) Immediate postoperative (day 0) radiograph demonstrating implant placement; (C) Early follow-up (day 14) showing maintained alignment; (D) Progressive healing (week 6th) with stable fixation; (E) Advanced healing (month 3) with callus formation; and (F) Late follow-up (9th month) showing complete healing and stable implant. Original radiographic images of the patient from the study, used with the patient's permission.

Representative serial radiographs illustrating postoperative implant positioning and progressive healing following surgical intervention are shown in Figure 1. The images demonstrated satisfactory alignment and stability of the prosthesis or fixation construct over time, with no radiographic evidence of implant failure or major complications, supporting the favorable clinical outcomes observed across surgical groups.

Longitudinal analysis of functional recovery showed a statistically significant improvement in the Functional Ambulation Category scores over time (Table 4). Functional ambulation improved progressively from the immediate postoperative period to one year across all fracture types, with no significant differences between the groups, indicating similar recovery trajectories irrespective of fracture location. A comparable trend was observed across the different surgical interventions (Table 5), with all groups demonstrating significant functional gains without intergroup differences.

**Table 4:** Functional ambulation category scores across follow-up intervals by fracture type. Values are expressed as mean ± standard deviation. Mixed-model repeated-measures analysis of variance (ANOVA) was used to assess changes between groups over time. Functional ambulation category scores range from 0 to 5, with higher scores indicating better ambulation. *p < 0.05 denotes statistical significance.

Follow‑up interval	Overall (Mean ± SD)	Femoral Neck (Mean ± SD)	Intertrochanteric (Mean ± SD)	Subtrochanteric (Mean ± SD)	Time (F value/p-value)	Intervention (F value/p-value)	Time x Intervention (F value/p-value)
1 day	0.14 ± 0.42	0.13 ± 0.34	0.14 ± 0.43	0.11 ± 0.33	528.4/0.001*	0.92/0.40	0.74/0.62
14 days	0.76 ± 0.81	0.72 ± 0.69	0.78 ± 0.84	0.67 ± 0.71			
6 weeks	1.63 ± 1.37	1.60 ± 1.29	1.64 ± 1.39	1.56 ± 1.42			
3 months	2.96 ± 1.39	2.91 ± 1.33	2.98 ± 1.41	2.89 ± 1.45			
6 months	3.77 ± 1.39	3.78 ± 1.37	3.77 ± 1.41	3.67 ± 1.41			
9 months	4.24 ± 1.12	4.23 ± 1.09	4.24 ± 1.12	4.32 ± 2.12			
1 year	4.58 ± 0.79	5.00 ± 0.00	4.50 ± 0.85	4.35 ± 1.54			

**Table 5:** Functional ambulation category scores across follow-up intervals by surgical intervention. Values are expressed as mean ± standard deviation. Mixed-model repeated-measures analysis of variance (ANOVA) was used for longitudinal comparison between interventions. No statistically significant differences were observed between intervention groups (p > 0.05).

Follow‑up interval	Overall (Mean ± SD)	Closed reduction and internal fixation with proximal femoral nailing (Mean ± SD)	Open reduction and internal fixation with proximal femoral nailing (Mean ± SD)	Bipolar Hemiarthroplasty (Mean ± SD)	Total hip replacement (Mean ± SD)	Time (F value/p-value)	Intervention (F value/p-value)	Time x Intervention (F value/p-value)
1 day	0.14 ± 0.42	0.15 ± 0.44	0.00 ± 0.00	0.10 ± 0.31	0.17 ± 0.41	512.8/0.001*	1.58/0.19	1.09/0.36
14 days	0.76 ± 0.81	0.79 ± 0.84	0.25 ± 0.50	0.70 ± 0.70	0.83 ± 0.75			
6 weeks	1.63 ± 1.37	1.64 ± 1.39	1.25 ± 1.50	1.63 ± 1.30	1.67 ± 1.37			
3 months	2.96 ± 1.39	2.99 ± 1.41	2.25 ± 1.50	2.93 ± 1.34	3.00 ± 1.41			
6 months	3.77 ± 1.39	3.76 ± 1.41	3.33 ± 1.53	3.88 ± 1.36	3.67 ± 1.37			
9 months	4.24 ± 1.12	4.24 ± 1.12	4.12 ± 1.32	4.29 ± 1.11	4.30 ± 1.23			
1 year	4.58 ± 0.79	4.50 ± 0.85	4.87 ± 1.43	5.00 ± 0.00	4.76 ± 1.87			

Analysis of the Modified Harris Hip Score also revealed significant improvement over time (Table 6), reflecting progressive enhancement in hip function across all fracture types. No statistically significant differences were observed between the fracture groups. Similarly, the evaluation across surgical interventions (Table 7) demonstrated comparable functional outcomes, indicating that the type of surgical procedure did not significantly influence long-term hip function.

**Table 6:** Modified Harris Hip Score across follow-up intervals by fracture type. Values are expressed as mean ± standard deviation. Mixed-model repeated-measures analysis of variance (ANOVA) was used to assess changes over time between types of fractures. Functional ambulation category scores range from 0 to 5, with higher scores indicating better ambulation. *p < 0.05 denotes statistical significance.

Follow‑up interval	Overall (Mean ± SD)	Femoral Neck (Mean ± SD)	Intertrochanteric (Mean ± SD)	Subtrochanteric (Mean ± SD)	Time, (F value/p-value)	Intervention, (F value/p-value)	Time x Intervention, (F value/p-value)
1 day	4.72 ± 7.59	5.00 ± 6.11	4.58 ± 7.98	5.56 ± 7.26	405.2/0.001*	2.08/0.13	1.38/0.18
14 days	23.50 ± 11.62	24.13 ± 10.75	23.18 ± 12.03	25.56 ± 10.24			
6 weeks	37.43 ± 13.90	38.75 ± 12.97	36.83 ± 14.24	40.00 ± 12.99			
3 months	52.60 ± 15.93	54.07 ± 15.05	51.92 ± 16.31	55.56 ± 14.67			
6 months	66.21 ± 11.58	67.61 ± 10.92	65.76 ± 11.83	66.00 ± 11.40			
9 months	73.10 ± 14.14	75.00 ± 12.87	72.50 ± 14.37	70.00 ± 10.43			
1 year	81.08 ± 4.46	83.50 ± 2.12	80.50 ± 4.74	79.87 ± 12.98			

**Table 7:** Modified Harris Hip Score across follow-up intervals by surgical intervention. Values are expressed as mean ± standard deviation. Mixed-model repeated-measures analysis of variance (ANOVA) was used to assess changes over time. Functional ambulation category scores range from 0 to 5, with higher scores indicating better ambulation. *p < 0.05 denotes statistical significance.

Follow‑up interval	Overall (Mean ± SD)	Closed reduction and internal fixation with proximal femoral nailing (Mean ± SD)	Open reduction and internal fixation with proximal femoral nailing (Mean ± SD)	Bipolar Hemiarthroplasty (Mean ± SD)	Total hip replacement (Mean ± SD)	Time, (F value/p-value)	Intervention, (F value/p-value)	Time x Intervention, (F value/p-value)
1 day	4.72 ± 7.59	4.63 ± 8.04	5.00 ± 5.77	5.00 ± 6.32	5.00 ± 5.48	398/0.001*	1.76/0.16	1.21/0.24
14 days	23.50 ± 11.62	23.14 ± 11.89	20.00 ± 11.55	25.00 ± 10.80	25.00 ± 10.95			
6 weeks	37.43 ± 13.90	36.94 ± 14.28	35.00 ± 15.00	38.75 ± 12.94	40.00 ± 12.65			
3 months	52.60 ± 15.93	52.07 ± 16.27	47.50 ± 16.58	54.33 ± 15.08	55.00 ± 14.72			
6 months	66.21 ± 11.58	65.75 ± 11.83	60.00 ± 13.23	68.10 ± 10.92	67.67 ± 10.97			
9 months	73.10 ± 14.14	72.50 ± 14.37	71.00 ± 12.43	75.00 ± 13.69	72.67 ± 11.98			
1 year	81.08 ± 4.46	80.50 ± 4.74	81.00 ± 14.76	85.00 ± 0.00	80.65 ± 12.56			

Assessment of culturally relevant functional outcomes demonstrated a gradual improvement in the ability to squat and sit cross-legged over time. No significant differences were observed during the early follow-up period; however, statistically significant differences emerged at nine months and one year when analyzed by surgical intervention (Table 8). Patients treated with closed reduction and internal fixation with proximal femoral nailing exhibited relatively less restoration of these functions than the arthroplasty groups at later follow-up intervals.

**Table 8:** Ability to squat and sit cross-legged by surgical intervention. Values are expressed as numbers (percentages), Fisher exact test was used for comparison. *p < 0.05 denotes statistical significance.

Follow‑up interval	Ability	Closed reduction and internal fixation with proximal femoral nailing (n=121)	Open reduction and internal fixation with proximal femoral nailing (n=4)	Bipolar Hemiarthroplasty (n=26)	Total hip replacement (n=6)	Chi stats	p-value
1 day	Unable	121 (100%)	4 (100%)	26 (100%)	6 (100%)	0.011	1.000
	With difficulty	0 (0%)	0 (0%)	0 (0%)	0 (0%)		
	With ease	0 (0%)	0 (0%)	0 (0%)	0 (0%)		
14 days	Unable	121 (100%)	4 (100%)	26 (100%)	6 (100%)	0.001	1.000
	With difficulty	0 (0%)	0 (0%)	0 (0%)	0 (0%)		
	With ease	0 (0%)	0 (0%)	0 (0%)	0 (0%)		
6 weeks	Unable	119 (98.3%)	4 (100%)	24 (92.3%)	6 (100%)	3.12	0.370
	With difficulty	2 (1.7%)	0 (0%)	2 (7.7%)	0 (0%)		
	With ease	0 (0%)	0 (0%)	0 (0%)	0 (0%)		
3 months	Unable	112 (92.6%)	4 (100%)	26 (100%)	6 (100%)	3.45	0.330
	With difficulty	9 (7.4%)	0 (0%)	0 (0%)	0 (0%)		
	With ease	0 (0%)	0 (0%)	0 (0%)	0 (0%)		
6 months	Unable	102 (84.3%)	3 (75.0%)	26 (100%)	6 (100%)	6.88	0.070
	With difficulty	19 (15.7%)	1 (25.0%)	0 (0%)	0 (0%)		
	With ease	0 (0%)	0 (0%)	0 (0%)	0 (0%)		
9 months	Unable	112 (92.6%)	3 (75.0%)	19 (73.1%)	3 (50.0%)	14.52	0.024*
	With difficulty	4 (3.3%)	1 (25.0%)	7 (26.9%)	3 (50.0%)		
	With ease	5 (4.1%)	0 (0%)	0 (0%)	0 (0%)		
1 year	Unable	91 (75.2%)	3 (75.0%)	18 (69.2%)	2 (33.3%)	16.03	0.014*
	With difficulty	16 (13.2%)	0 (0%)	5 (19.2%)	4 (66.7%)		
	With ease	14 (11.6%)	1 (25.0%)	3 (11.5%)	0 (0%)		

When analyzed according to fracture type (Table 9), significant differences were observed at intermediate follow-up intervals, with progressive recovery noted across all groups. Femoral neck fractures demonstrated relatively better functional improvement at later stages, suggesting the potential influence of fracture location on the recovery of culturally relevant functional activities.

**Table 9:** Ability to squat and sit cross-legged by fracture type. Values are expressed as numbers (percentages), Fisher exact test was used for comparison. *p < 0.05 denotes statistical significance.

Follow‑Up Interval	Ability	Femoral Neck (n = 32)	Intertrochanteric (n = 119)	Subtrochanteric (n = 9)	Chi-stat	P‑Value
1 Day	Unable	32 (100%)	119 (100%)	9 (100%)	0.011	0.684
	With Difficulty	0 (0%)	0 (0%)	0 (0%)		
	With Ease	0 (0%)	0 (0%)	0 (0%)		
14 Days	Unable	32 (100%)	118 (99.1%)	8 (88.9%)	0.001	0.999
	With Difficulty	0 (0%)	01 (0.9%)	1 (11.1%)		
	With Ease	0 (0%)	0 (0%)	0 (0%)		
6 Weeks	Unable	32 (100%)	116 (97.3%)	7 (77.8%)	7.82	0.020*
	With Difficulty	0 (0%)	2 (1.8%)	2 (22.2%)		
	With Ease	0 (0%)	1 (0.9%)	0 (0%)		
3 Months	Unable	32 (100%)	112 (93.7%)	6 (66.7%)	15.64	<0.001*
	With Difficulty	0 (0%)	6 (5.4%)	2 (22.2%)		
	With Ease	0 (0%)	1 (0.9%)	1 (11.1%)		
6 Months	Unable	32 (100%)	101 (84.9%)	7 (77.8%)	14.21	<0.001*
	With Difficulty	0 (0%)	17 (14.3%)	1 (11.1%)		
	With Ease	0 (0%)	1 (0.8%)	1 (11.1%)		
9 Months	Unable	22 (68.8%)	100 (84.0%)	7 (77.8%)	5.12	0.080
	With Difficulty	9 (28.1%)	17 (14.3%)	1 (11.1%)		
	With Ease	1 (3.1%)	2 (1.7%)	1 (11.1%)		
1 Year	Unable	22 (68.8%)	100 (84.0%)	6 (66.7%)	6.01	0.050
	With Difficulty	8 (25.0%)	17 (14.3%)	2 (22.2%)		
	With Ease	2 (6.2%)	2 (1.7%)	1 (11.1%)		

## Discussion

The present study evaluated the functional outcomes following hip fracture surgery in elderly patients in a rural hilly tertiary care setting. The findings demonstrated that although delays in surgical intervention were common, postoperative complications were minimal, survival rates were high, and significant functional recovery was achieved over time, irrespective of fracture type or surgical modality.

The demographic profile of the study population showed a predominance of elderly females, which is consistent with the well-established higher incidence of osteoporotic fractures among women. Similar trends have been reported by Salari et al. [9], who highlighted the increased lifetime risk of hip fractures among females owing to reduced bone mineral density and postmenopausal changes. The high proportion of intertrochanteric fractures observed in this study is consistent with previous epidemiological studies, which suggest that these fractures are more common in elderly individuals owing to the biomechanical vulnerability of the trochanteric region [10].

A notable finding of this study was the delay in surgical intervention, with the majority of patients undergoing surgery beyond six days of admission. This contrasts with international guidelines recommending surgery within 24-48 hours to reduce morbidity and mortality. Smektala et al. [11] demonstrated that delayed surgery was associated with increased complications and mortality. However, despite surgical delays in the present study, the complication rates remained low, and survival outcomes were favorable. This discrepancy may be attributed to the effective postoperative management, patient selection, or relatively stable preoperative conditions in the study population.

The present study found no statistically significant differences in postoperative outcomes across fracture types or surgical interventions. This suggests that factors such as fracture location and choice of surgical procedure may not independently influence short-term clinical outcomes when appropriate management protocols are followed [12]. Similar observations were reported by Roberts et al. [13], who emphasized that patient-related factors and postoperative care play a more critical role than fracture type alone in determining outcomes.

Functional recovery showed significant improvement over time in all groups. These findings are consistent with those of Koval et al. [14] and Takahashi et al. [8], who demonstrated progressive recovery of mobility and hip function following surgical intervention. Importantly, no significant differences were observed between the fracture types or surgical modalities, indicating that both internal fixation and arthroplasty can achieve comparable functional outcomes when appropriately indicated. Buecking et al. [15] demonstrated that early postoperative mobilization following hip fracture surgery is influenced by multiple factors, including patient comorbidities, cognitive status, and postoperative care. Their findings emphasize that timely rehabilitation and optimized clinical management play crucial roles in improving functional recovery during the early postoperative period.

In the present study, comparable functional outcomes were observed across different fracture types and surgical interventions at one year. However, it is important to note that the choice of treatment modality was primarily dictated by fracture pattern, geometry, and individual patient factors, making direct comparisons between groups inherently challenging. Internal fixation techniques, including osteosynthesis, preserve the native femoral head and bone stock, which may contribute to favorable long-term functional outcomes. Additionally, these procedures are often associated with shorter operative duration, reduced hospital stay, and lower transfusion requirements compared to arthroplasty. Therefore, treatment strategies should be individualized based on fracture characteristics and patient-specific considerations rather than direct comparison of surgical modalities.

However, when culturally relevant functional outcomes, such as squatting and sitting cross-legged, were evaluated, significant differences emerged at later follow-up intervals. Patients treated with internal fixation, particularly closed reduction and internal fixation with proximal femoral nailing, demonstrated relatively less recovery of these functions than the arthroplasty groups. Kumar et al. [16] demonstrated that proximal femoral nailing provides superior functional outcomes compared to hemiarthroplasty in elderly patients with unstable intertrochanteric fractures. Their meta-analysis highlighted improved mobility and reduced complication rates with internal fixation, supporting its preference for appropriate clinical scenarios. Prakash et al. [17] reported that proximal femoral nailing demonstrated better functional outcomes and earlier mobilization than dynamic hip screw fixation in patients with intertrochanteric femur fractures. The study also highlighted improved biomechanical stability and reduced complications associated with intramedullary fixation, supporting its use in unstable fracture patterns.

Interestingly, fracture type appeared to influence the recovery of culturally specific functions, with femoral neck fractures demonstrating comparatively better outcomes in later stages. This may be attributed to differences in biomechanics, surgical techniques, or rehabilitation potentials. However, further studies are required to validate these observations. The radiographic findings in the present study demonstrated satisfactory implant positioning and progressive healing without evidence of major complications, thus supporting the favorable clinical outcomes observed. These findings reinforce the importance of proper surgical techniques and postoperative care in achieving optimal outcomes.

Clinical implications

The findings of this study have important clinical implications, particularly in resource-limited and geographically challenging settings. Despite delays in surgical intervention, favorable outcomes can be achieved with appropriate postoperative management and rehabilitation. This study also highlights that the choice of surgical intervention should be individualized based on patient characteristics rather than fracture type alone. Furthermore, the assessment of culturally relevant functional outcomes is essential in evaluating true recovery, especially in populations where activities such as squatting and sitting cross-legged are integral to daily life. Internal fixation techniques may offer advantages in restoring these functions and should be considered when feasible.

Limitations

This study had certain limitations. First, it was conducted at a single tertiary care center, which may limit its generalizability. Second, although the overall sample size was adequate, the subgroup analysis for certain surgical interventions had smaller and unequal numbers, which may have affected the statistical power and intergroup comparisons. Third, the delay in surgical intervention was not controlled for and may have introduced confounding factors. Additionally, the observational design did not allow adjustment for important potential confounders such as comorbidities and baseline functional status, which may have influenced the outcomes. In addition, long-term outcomes beyond one year were not assessed. Finally, the rehabilitation protocols were not standardized, which could have influenced functional recovery.

## Conclusions

Hip fracture surgery in elderly patients resulted in significant improvement in functional outcomes over time, irrespective of the fracture type or surgical modality. Despite delays in surgical intervention, the complication rates were low, and survival outcomes were favorable. Both internal fixation and arthroplasty provided comparable overall functional recovery; however, arthroplasty demonstrated better restoration of culturally relevant activities, such as squatting and sitting cross-legged. These findings highlight the importance of individualized treatment planning and emphasize the need to incorporate culturally specific functional outcomes when evaluating recovery in the Indian population.
